# Specific, reversible G1 arrest by UCN-01 in vivo provides cytostatic protection of normal cells against cytotoxic chemotherapy in breast cancer

**DOI:** 10.1038/s41416-019-0707-z

**Published:** 2020-01-16

**Authors:** Benjamin B. Mull, J. Andrew Livingston, Nalini Patel, Tuyen Bui, Kelly K. Hunt, Khandan Keyomarsi

**Affiliations:** 1 Department of Experimental Radiation Oncology, The University of Texas MD Anderson Cancer Center, Houston, TX USA; 2 Department of Sarcoma Medical Oncology, The University of Texas MD Anderson Cancer Center, Houston, TX USA; 3 Department of Breast Surgical Oncology, The University of Texas MD Anderson Cancer Center, Houston, TX USA

**Keywords:** Breast cancer, Cell-cycle exit

## Abstract

**Background:**

Low-dose UCN-01 mediates G1 arrest in normal proliferating cell lines with an intact G1 to S transition but not tumour cells with a deregulated G1 to S checkpoint. Here we hypothesised that UCN-01 is effective in mediating a selective, reversible G1 arrest of normal proliferating cells, resulting in decreased chemotoxicity, improved tolerance and enhanced chemotherapeutic efficacy in vivo in both non-tumour-bearing mice and in breast cancer cell line xenograft models.

**Methods:**

Murine small bowel epithelium was used to examine the kinetics and mechanism of low-dose UCN-01-mediated arrest of normal proliferating cells and if it can protect tumour-bearing mice (MDA-MB-468 xenografts) against the toxic effects of chemotherapy (5-fluorouricil (5-FU)) allowing for its full therapeutic activity.

**Results:**

UCN-01 causes significant, reversible arrest of normal gut epithelial cells at 24 h; this arrest persists for up to 7 days. Normal cellular proliferation returns by 2 weeks. Pre-treatment of both non-tumour-bearing and MDA-MB-468 tumour-bearing mice with UCN-01 prior to bolus 5-FU (450 mg/kg) yielded enhanced therapeutic efficacy with significantly decreased tumour volumes and increased survival.

**Conclusions:**

UCN-01 mediates a specific, reversible G1 arrest of normal cells in vivo and provides a cytoprotective strategy that decreases toxicity of cytotoxic chemotherapy without compromising efficacy.

## Background

Despite significant advances in molecular cancer therapeutics and cancer immunotherapy, cytotoxic chemotherapy remains the mainstay of treatment for most cancer patients in the neoadjuvant, adjuvant, or metastatic setting. The toxic effects of chemotherapy are nonspecific, often affecting healthy proliferating cells, such as the gut epithelium and hematopoietic precursors, which can result in dose-limiting toxicities. Strategies that can exploit the differential regulation of normal cells as compared with tumour cells to protect normal cells from chemotoxicity may allow for dose escalation, enhance the therapeutic window for currently available cytotoxic chemotherapies, and reduce the development of resistance.^1,2^

To be effective, these cytoprotective strategies^3^ must fulfil two major criteria: (1) cell cycle arrest must be specific to normal proliferating cells without affecting tumour cells, and (2) this arrest of normal cells must be reversible. Our lab has previously shown that differences in cell cycle regulation, specifically the G1 checkpoint, can be exploited in a two-step strategy to protect normal cells while maintaining chemotherapeutic efficacy against tumour cells in vitro.^4,5^ First, the normal proliferating cells are blocked in the G0/G1 phase of the cell cycle by pre-treatment with cytostatic agents such as staurosporine or UCN-01, at very low doses specific to normal proliferating cells due to their intact G0/G1 checkpoint. Tumour cells will not respond to these agents at low dose, due to loss of the G0/G1 checkpoint, and continue to proliferate. Following removal of the cytostatic agents, both normal and tumour cells are treated with conventional chemotherapeutic agents, which are cytotoxic to proliferating tumour cells. Normal cells are protected because of the G0/G1-mediated reversible arrest achieved in the first step. However, tumour cells, which were unresponsive to the protective agent, continue to proliferate and undergo chemotherapy-mediated cell death.

UCN-01 (7-hydroxystaurosporine) is an indolocarbazole compound originally isolated from a culture broth of *Streptomyces* sp. and initially developed as a selective protein kinase C (PKC) inhibitor.^6^ Subsequently, studies have shown it to be a nonselective tyrosine kinase inhibitor (TKI) with multiple additional targets, including cAMP dependent protein kinase and v-src tyrosine kinase, among others.^7,8^ As a single agent, UCN-01 can either arrest cells in the G1 phase of the cell cycle or drive apoptosis, depending on the dose that is used and/or the G0/G1 checkpoint status of the cells. Its ability to produce cell cycle arrest may be mediated, at least in part, by the Rb status of the cells being treated, with wild-type Rb permitting G1 arrest (low doses) and lack of Rb resulting in apoptosis (high doses).^9,10^ In vitro, in responsive cells, G1 arrest at low doses is accompanied by decreases in CDK2 and CDK4, as well as CDK2-associated kinase activities; Rb phosphorylation decreases concomitant with arrest as does cyclin D.^5^ Additionally, UCN-01 can affect E2F-mediated transcription of G1- and S-phase genes by suppressing E2F protein levels.^11^ UCN-01 treatment of normal cells results in signification inhibition of proliferation of bone marrow cells and the small bowel gut epithelium.^12^ Treatment of normal cells with concentrations of UCN-01 that are significantly below the drug’s anti-tumour efficacy doses, can result in reversible G1 arrest in normal cells, but does not inhibit growth in tumour cells. Capitalising on the defective G1 checkpoint in many cancer cells, low dose UCN-01 is able to specifically arrest normal cells in culture, while tumour cell lines are unaffected and continue to proliferate. The differing sensitivity to low dose UCN-01 in vitro is dependent on functional pRb and intact G1 checkpoint regulation.^5^

In this study, we explore the in vivo use of UCN-01 to protect normal dividing cells in mice from the toxicity of chemotherapy. Previously, we have shown that cells with wild-type Rb can be arrested in G1 with low dose UCN-01 in vitro and that these cells return to a proliferative state once the drug has been removed, but these effects have not previously been examined in vivo.^5^ We hypothesised that low dose UCN-01 would be effective in mediating a selective, reversible G1 arrest, resulting in decreased chemotoxicity, improved tolerance and possibly enhanced chemotherapeutic efficacy. Using a mouse small bowel epithelium model system, we evaluated various dosing strategies to optimise the efficacy of UCN-01 as a cytoprotective agent and characterised the molecular changes associated with cell cycle arrest of normal cells in vivo. Subsequently, we applied these findings to a tumour-bearing mouse model using pre-treatment with UCN-01 as a cytoprotective strategy.

## Methods

### Mice

Female NU/NU immunodeficient nude mice from 8–12 weeks of age (~24–30 g) were obtained from the Experimental Radiation Oncology colony at MD Anderson Cancer Center. Mice received care in accordance with the Animal Welfare Act and the institutional guidelines of MD Anderson Cancer Center. The protocol for this study was approved by the Institutional Animal Care and Use Committee (IACUC) at The University of Texas MD Anderson Cancer Center (Houston, TX).

#### UCN-01/mock treatment

Mice were injected with UCN-01 or carrier (dimethyl sulfoxide, DMSO, Sigma Aldrich, St. Louis MO) intramuscularly (i.m.) into the right hind limb. UCN-01 was obtained from the NCI Chemotherapeutic Agents Repository and is re-suspended in DMSO at 7.5 mg/ml. Prior to injection, the UCN-01 solution is diluted 2:1 with sterile normal saline for a final concentration of 5 mg/ml. DMSO given as vehicle control was diluted in the same fashion and was given as a volume equivalent dose. To obtain the normal mouse values for flow cytometry, mice either received no treatment or were given volume equivalent PBS in the right hind limb.

#### Fluorouracil treatment

Mice were given 5-FU (50 mg/ml, Abraxis Pharmaceuticals, Schaumburg IL) intraperitoneally (i.p.) via a 0.5 ml insulin syringe with a 32-gauge needle. Mice were treated with either a single bolus dose or five daily injections, depending on the experimental protocol.

### Tumour xenografts

Nu/nu mice were given 2% isoflurane to anesthetise the mice for 5 min, which was confirmed with lack of toe pinch withdrawal reflex. In all, 5 × 10^6^ MDA-MB-436 cells were suspended in 100 µL of media and injected subcutaneously 5 mm from the 4th nipple into the mammary fat pad of each of the nu/nu female mice and observed for any complications. Mice were treated with UCN-01 or DMSO control followed by treatment with 5-FU or PBS control, similar to the treatment of non-tumour-bearing mice. Sustained release buprenorphine at 1.0 mg/kg was administered every 6–12 h by subcutaneous injection with a 28-gauge needle between shoulder blades for analgesia as needed for pain.

### Euthanasia

A Euthanex system (Euthanex Corporation, Palmer PA), operated by veterinary technicians, was used to euthanise mice. CO_2_ was provided using a CO_2_ tank with gauge and pressure regulator to displace 10–30% of the cage volume per minute. Mice were exposed for 5 min for complete cessation of breathing. Following CO_2_ exposure, mice were exposed to ambient air for 20–30 min to ensure non-recovery.

### Bromodeoxyuridine labelling

Mice were sacrificed at one of five time points following UCN-01/mock injection: 24 h, 48 h and 7, 14 or 28 days, and the tissue processed (Supplementary Fig. S1). On the day of sacrifice, mice were injected i.p. (as above for 5-FU) with 100 µl of 60 mg/kg BrdU (Sigma Aldrich, St. Louis MO) 6 h prior to sacrifice (as described under Euthanasia). Various time points post-BrdU injection were analysed for Bromodeoxyuridine (BrdU) staining and 6 h post-injection was determined to be optimal (Supplementary Fig. S1). Mice were staggered by injection and sacrifice times such that each mouse had exactly 6 h of BrdU exposure. Upon sacrifice, the jejunum was dissected out and flushed with ice-cold ethanol (60% in PBS, both Fisher Scientific, Pittsburgh, PA). A small piece was fixed in formalin (10%, Fisher Scientific), and the remainder fixed overnight in the 60% ethanol/PBS buffer. To prepare the tissues for flow cytometry, the jejunums were opened longitudinally and placed on a glass microscope slide. A second slide was dragged across the epithelial surface to dislodge the cells; care was taken not to apply excess pressure and disrupt the basement membrane. The isolated cells were re-suspended in 5 ml of 0.04% pepsin (Fluka chemical, obtained from Sigma Aldrich) in a 50 ml glass flask and placed in a shaking water bath at 37 °C at 90 Hz for 1 h. The cells were filtered through 35-μm mesh and centrifuged at 1200 rpm for 5 min in a swinging bucket centrifuge (Beckman Coulter, Brea CA). Cells were re-suspended in 1.5 ml of 2 N HCl (Fisher Scientific) for 20 min in a 37 °C incubator. The acid was neutralised with 3 ml 0.1 M sodium borate (Sigma Aldrich) and the cells were again centrifuged at 1200 rpm for 5 min. The cells were washed with 5 ml of PBTB (phosphate-buffered saline plus 0.5% Tween-20 (Sigma Aldrich) and 0.5% bovine serum albumin (Fisher Scientific) and centrifuged at 1200 rpm for 5 min. Samples were resuspended in primary anti-BrdU antibody (IU-4, Caltag, Burlingame CA) diluted 1:100 in PBT (phosphate-buffered saline plus 0.5% Tween-20) for 60 min. Samples were washed in 5 ml of PBTB and centrifuged at 1200 rpm for 5 min. Samples were resuspended in 0.2 ml of secondary antibody (goat anti-mouse fluorescein isothiocyanate (GAM-FITC), Beckman Coulter, Brea CA) diluted 1:100 in PBTG (PBT with 2% normal goat serum) for 60 min, then washed in PBTB and centrifuged at 1200 rpm for 5 min. Samples were then suspended in propidium iodide solution (1 mg/ml PI (Roche, Branford CT) in 95% ethanol diluted 1:100 in PBT) and stored at 4 °C overnight.

### Flow cytometry

Intestinal digests were analysed using a Beckman Coulter flow cytometer. DNA content (PI, *x*-axis) and BrdU-positive nuclei (log BrdU, *y*-axis) are counted and the results exported to the ModFit LT program (Verity software, Topsham ME). Labelling index is calculated by dividing the number of BrdU-positive nuclei by the total number of nuclei analysed. The fraction of labelled divided cells (f^ld^) is the number of BrdU-positive nuclei which are in G1 phase divided by the total number of nuclei counted. The fraction of labelled undivided cells (f^lu^) is the number of BrdU-positive nuclei which correspond to the S and G2/M phases of the cell cycle divided by the total number of cells analysed. A complete explication of the flow analysis parameters and other values which may be calculated can be found in a methods article.^13^

### Immunohistochemistry

Jejunum sections were embedded in paraffin and 5 μM sections were cut onto superfrost/plus slides (Fisher Scientific). To visualise BrdU in the sections, slides were processed as for IHC. Slides were hydrated in three washes of xylene (Fisher Scientific) for 5 min each, and then 3 min each of 100%, 90 and 70% ethanol. Slides were washed for 5 min in water, and then digested in 0.1% protease (Sigma Aldrich) for 1 h. Slides were washed twice for 5 min in PBS and placed into 2 N hydrochloric acid for 50 min. The acid was neutralised in 0.1 M sodium borate. Slides were washed twice in PBST (PBS plus 0.5% Tween 20, Sigma Aldrich) for 5 min and then blocked in normal horse serum (Vector Labs, 3 drops in 10 ml PBST) at 37º for 30 min in a humidified chamber. The slides were incubated with primary IU-4 antibody (1:100 in PBST) for 1 h in a humidified chamber at 37 °C. After two five-minute washes in PBST, the slides were incubated with secondary GAM-FITC (1:100 in PBST plus 1% normal goat serum) for 1 h at 37 °C in a dark humidified chamber. After two washes of 5 min each in PBST, the nuclei were stained with 0.1 μg/ml Propidium iodide (Sigma Aldrich) for 15 min. Slides were mounted in antifade medium (Vectasheild, Vector Labs, Burlingame CA) and sealed with a glass coverslip.

### Microscopy

Stained jejunum sections were visualised using a Leica DM400B (Wetzlar, Germany) fluorescent microscope. Images were taken using a Spot digital camera and Spot Advanced software (Spot Imaging Solutions, Sterling Heights MI).

Western blot analyses were performed in standard fashion as previously described.^14^

Serial fractionation of the gut epithelium were performed as previously described.^15,16^ Briefly, the jejunum was dissected out of the abdomen and flushed with 10 ml ice-cold PBS, followed by 10 ml ice-cold PBS plus 1 mM dithiothreitol (DTT). The tissue was then tied closed on one end with 3–0 silk suture (Ethicon/Johnson & Johnson, Warsaw IN) and then everted. The everted tissue was filled with ice-cold PBS to distension, tied closed followed by incubation in a citrate buffer (96 mM NaCl, 1.5 mM KCl, 27 mM Na citrate, 8 mM KH_2_PO_4_ and 5.6 mM Na_2_HPO_4_, pH 7.3) for 15 min at 37 °C. The tissue was then placed in a 50 ml glass flask in 15 ml of a PBS buffer with 1.5 mM EDTA, 0.5 mM DTT and 1 mg/ml bovine serum albumin (BSA). The flask is placed in a 37 °C water bath shaking at 90 Hz for 25 min. The buffer with dislodged cells is collected and spun down at 1100 rpm for 5 min at 4 °C; the cell pellet is washed with 10 ml ice-cold PBS, spun down again and resuspended protease/phosphatase inhibition buffer: [25 µg/ml leupeptin, 25 µg/ml aprotinin, 10 µg/ml pepstatin, 1 mM benzamidine, 10 µg/ml soybean trypsin inhibitor, 0.5 mM PMSF (Phenyl methyl sulfonyl fluoride), 50 mM sodium fluoride, 0.5 mM sodium orthovanadate] and processed as above and was designated as fraction 1. The tissue was then placed in a new flask and shaken at 37 °C for an additional 35 min; the cells are processed as before and designated as 2. A third incubation for 60 min is collected and processed and designated as fraction 3.

### Statistical analysis

Pairwise comparison of means was performed using Student’s *t*-test; variation determination among and between multiple experimental conditions was performed using analysis of variance (ANOVA); confidence level of 95% was considered to be statistically significant in these studies. Survival analysis was interpreted using the Kaplan-Meier method and significance for comparing treatment outcomes was performed using the Mantel-Cox Log Rank test. All calculations were performed using the Prism software package (GraphPad Software,Inc.).

## Results

### UCN-01 reversibly arrests normal gut epithelium in G1

**Fig. 1:**
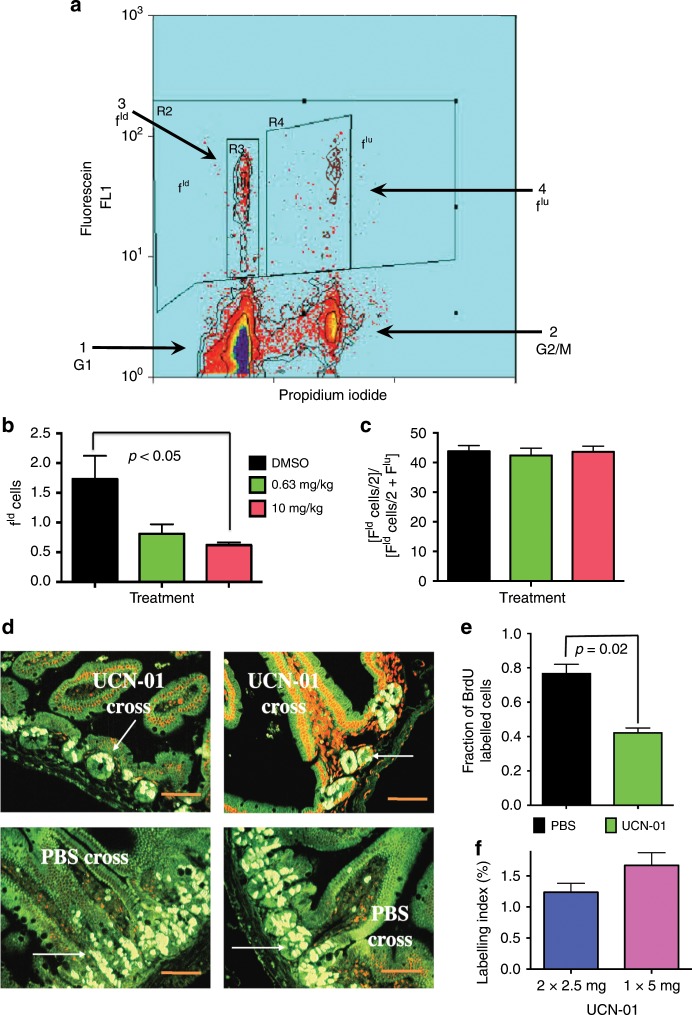
UCN-01 arrests the proliferating cells of nu/nu nude mouse small intestine. **a** Bivariate analysis of mouse jejunum BrdU labelling allows for identification of G1/G0 cells (1), G2 (2), the fraction of labelled divided cells (f^ld^, 3) which are BrdU positive and have gone through mitosis, and the fraction of labelled undivided cells (f^lu^, 4) which have yet to divide; **b** the fraction of labelled divided cells (f^ld^) was significantly decreased 48 h after treatment with UCN-01 at 10 mg/kg; **c** No difference was observed in the percentage of BrdU-labelled cells following treatment with UCN-01, suggesting that the decrease in f^ld^ reflects a decrease in cells entering S phase rather than a block in G2/M phase; **d** IHC of jejunal sections (left and right panels refer to two biological replicates with the same dose) shows diminished levels of BrdU incorporation in UCN-01 treated mice (top two panels) as compared with the PBS treated mice (bottom two panels); **e** the quantitation of fraction BrdU-labelled cells from **d**. **e** the degree of cell cycle arrest was comparable whether UCN-01 was administered as a single or split dose. Scale bar: ×200 magnification.

To demonstrate the ability of UCN-01 to arrest normally dividing cells in vivo, we examined the epithelium of the small intestine of nu/nu nude mice. This is a rapidly dividing tissue, in which cells are continually replicating in the crypt to replace the cells at the villus tip as they die and are shed into the lumen. Cell cycle kinetics in the mouse small bowel were analysed by flow cytometry to identify changes following UCN-01 treatment. Bivariate analysis allowed for identification of cells in each phase of the cell cycle; the fraction of labelled divided cells (f^ld^), which are BrdU-positive cells that have gone through mitosis, and the fraction of labelled undivided cells (f^lu^), correspond to the BrdU-positive cells which have yet to divide (Fig. 1a). To establish arrest, nu/nu nude mice were injected with either DMSO or UCN-01 at 0.63 mg/kg or 10 mg/kg. Mice were injected with BrdU at 48 h, jejunum was harvested 6 h post-injection, and growth arrest was analysed by flow cytometry. Both doses of UCN-01 resulted in arrest of proliferating cells compared with DMSO controls, although this was not statistically significant at the lower dosage (*p* = < 0.05 at 10 mg/kg, 0.0516 at 0.63 mg/kg, Fig. 1b). The overall percentage of BrdU-labelled cells was the same in all groups, an indication that the decrease in proliferation is not due to a G2/M block, but rather a decrease in cells entering S phase (Fig. 1c). Initial experiments using DMSO as a control suggest that the vehicle stimulates proliferation of mouse intestinal epithelium, consistent with prior studies.^17^ Therefore, a baseline value f^ld^ value for untreated mice was established for future experiments with untreated mice vs. PBS IM injection at 48 h. Flow cytometric analysis showed no difference in f^ld^ cells between the untreated and PBS groups (*p* *=* n.s,. mean f^ld^ value for all mice 0.99%, data not shown). Next, mice were treated with either 5 mg/kg UCN-01 or PBS for 24 h, followed by BrdU injection. Immunofluorescence visualisation of BrdU, in which BrdU incorporation into a cell indicates active cell proliferation, showed significantly lower BrdU incorporation in UCN-01-treated samples as compared with control, consistent with diminished cellular proliferation (Fig. 1d, e). We next examined whether splitting the dose of UCN-01 over a 24-hour period would enhance the reduction of cell proliferation. This alternate dose strategy (UCN-01 2.5 mg × 2 doses, 12 h apart) did demonstrate further reduction in cellular proliferation but was not statistically significant. Both the single-dose and split-dose regimens were highly effective and resulted in a significant reduction of cell proliferation (Fig. 1f). Thus, a single dose of 5 mg was chosen for future experiments. The dose of 5 mg/kg is considered a low dose of UCN-01 that is normal cell specific. For comparison, the maximal tolerated dose/recommended dose for short infusion UCN-01 from phase 1 clinical trial of UCN-01 in patients with solid tumours was 95 mg/m2, whereas the dose of 5 mg/kg in mice is ~15 mg/m2.^18,19^ Taken together, these studies confirm that UCN-01 is able to arrest the proliferating cells of nu/nu nude mouse small intestine.

**Fig. 2:**
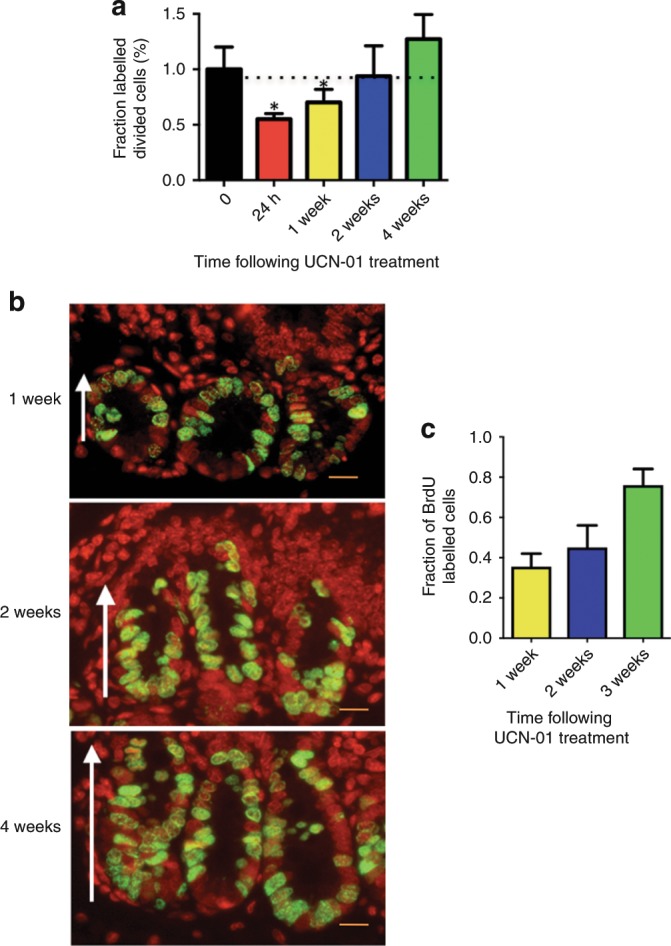
Arrest and recovery of mouse jejunal cells in vivo following UCN-01 treatment. **a** Arrest by flow cytometry occurs within 24 h of administration of UCN-01 at 5 mg/kg (*n* = 20), persists through 1 week (*n* = 20), returns to near normal/baseline by 2 weeks (dotted line) (*n* = 20), and gives way to slightly increased proliferation by week 4 (*n* = 20); **b** IHC for BrdU shows BrdU incorporation in jejunal crypt cells (green; nuclei stained in red) is lowest at 1 week post-UCN-01 treatment at 5 mg/kg and increases through weeks 2 and 4. White arrows indicate crypt to villus orientation. **c** Quantiation of fraction of BrdU-labelled cells from **b**. Scale bar: ×800 magnification.

Since one of the criteria for chemoprotection is reversibility of the arrest of the normal tissue, we sought to establish the reversibility and timeline to recovery from growth arrest of normal gut epithelium. Nu/nu nude mice were treated with 5 mg/kg UCN-01 or DMSO control, 40 mice per group. Mice were BrdU-labelled, and cohorts sacrificed at 24 h, 1 week, 2 weeks and 4 weeks post treatment (10 mice per time point, per group). Proliferation was again assessed by flow cytometry to identify the fraction of BrdU-labelled divided cells (f^ld^). The f^ld^ values for UCN-01-treated mice were lower than our established baseline at 24 h and 1-week post treatment, suggesting inhibition of jejunal proliferation. f^ld^ values increased to near baseline at 2 weeks and were slightly higher than baseline by at 4 weeks following UCN-01 treatment, suggesting reversibility of the UCN-01 mediated arrest (Fig. 2a). The experiment was repeated with another set of 40 mice per treatment group at 10 mice per group per time point (24 h, 1 week, 2 weeks and 4 weeks) and the data from the two experiments were combined and presented in Fig. 2a. Representative BrdU IHC and their quantiation reveals that fewer BrdU cells were observed at 1 week as compared with 2- and 4-weeks post treatment (Fig. 2b, c). Collectively, these studies suggest that gut epithelial cells are in a less proliferative state at 1 week as compared with 2 and 4 weeks following UCN-01 treatment, as less BrdU has been incorporated during S phase, consistent with the flow cytometry results. The increase in proliferation seen at 4 weeks was postulated to be a result of release from growth arrest caused earlier by UCN-01, in which greater cell proliferation is intended to repopulate the villi; this is similar to the response seen when mice are treated with γ-irradiation.^20^ Our results also suggest that treatment of mice with 5 mg/kg UCN-01 is sufficient to consistently (amongst the 20 mice per time point) cause a significant arrest of the gut epithelial cells as early as 24 h after treatment. This arrest persists through day 7 post-UCN-01 treatment, and the intestinal epithelia return to the normal level of proliferation by two weeks post-UCN-01 treatment. These findings suggest that in vivo, normal dividing cells of the mouse small bowel can be reversibly arrested by UCN-01 administration, fulfilling one major criterion for its use as a cytoprotective agent.

### UCN-01 treatment results in a post-mitotic G1 arrest of normal cells in vivo

**Fig. 3:**
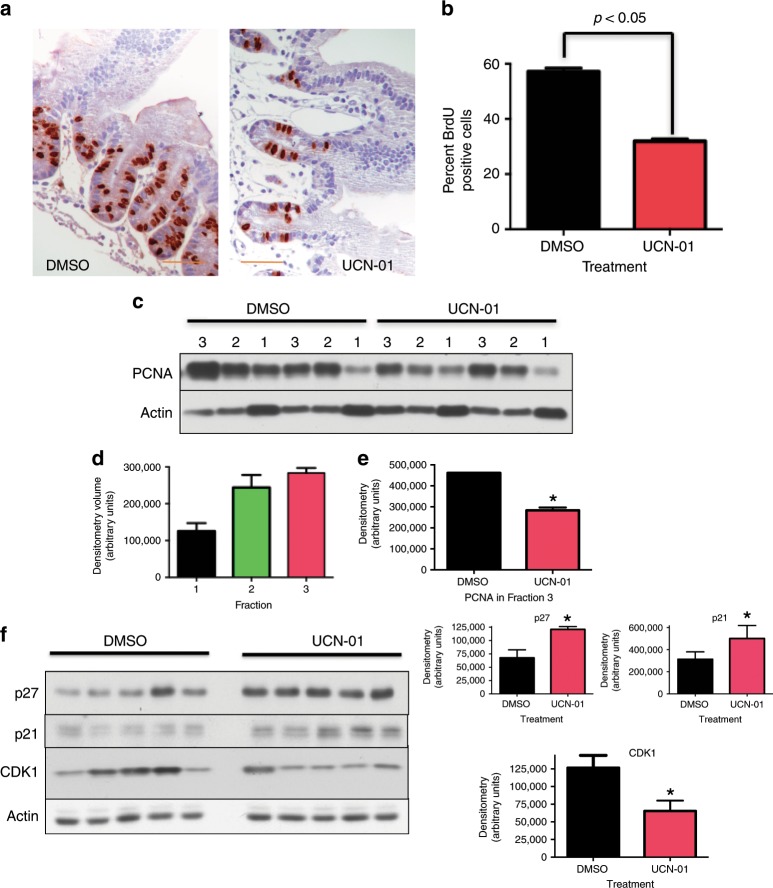
Analysis of key cell cycle regulators in jejunal crypt cells suggests post-mitotic G1 arrest by UCN-01. **a** Representative IHC for BrdU incorporation into the nuclei of mouse jejunum 24 h after DMSO or 5 mg/kg UCN-01; **b** UCN-01 treatment results in significant decrease in BrdU incorporation in jejunal crypt cells (five mice per group, 10 crypts per mouse analysed); **c** Serial fractions of jejunal epithelium were separated using the Weiser method (see Materials and Methods) This method uses heat and shaking everted intestinal section to detach ‘fractions’ of gut eothelial cells into buffered PBC solution over a 2 h period. The early fractions (i.e. 1 and 2) contain cells near the villus tip and while the late fraction (i.e. 3) are enriched in cells from the crypt region, Protein from four mice was collected in three fractions following 24, 36 and 60 of minutes of incubation respectively. Proliferation as measured by PCNA was low in fraction 1, increased in fraction 2, and highest in fraction 3, indicative of proliferative cells found in the crypt; **d** Densitometry of UCN-01 fractions indicates increased levels of PCNA in the crypt fraction (3) and is significantly lower than DMSO controls (**e**), indicating that UCN-01 treatment is effective in causing cell cycle arrest; **f** Key G1 proteins in the crypt fraction were analysed by western blot and showed significant induction in p21 and p27 with concurrent down regulation of CDK1 following UCN-01 treatment. Scale bar: ×400 magnification.

To investigate the modulation of the G0/G1 pathway UCN-01 and the role of Rb in normal tissue in vivo, we first examined the expression of phospho-Rb in the crypt versus the villi of the gut epithelium in the UCN-01 and DMSO treated mice. Mice were injected with either 5 mg/kg UCN-01 or DMSO and 24 h later were sacrificed and the jejunum harvested. Phosphorylation of Rb was assessed by phospho-specific anti-phospho-S807/S811-Rb antibody in mouse jejunum. Results revealed that phospho-Rb expression increased significantly only in the crypt cells, but not the cells in the villi in the UCN-01 treated mice compared with DMSO controls (Supplementary Fig. S2). To ensure that the cells were indeed arrested by UCN-01 treatment as in prior experiments, the proliferation status of the crypt cells was analysed by BrdU staining; a decrease in BrdU-positive crypts cells in the treatment group was consistent with decreased DNA synthesis in the UCN-01-treated mice (Fig. 3a, b). The combination of low levels of DNA synthesis (indicative of cell cycle arrest) and high levels of phospho-Rb seems to indicate that the cells exposed to UCN-01 are traversing the post-mitotic portion of G1 (thus phosphorylating Rb) but are not crossing the restriction point into S phase (leading to the diminished levels of BrdU incorporation).

To determine which key cell cycle regulators are required for UCN-01-mediated G1 arrest in normal cells in vivo, we examined the expression of several positive and negative cell cycle proteins in crypt cells within the murine small bowel epithelium. Because our area of interest in the crypt/villus architecture in the gut is the small proliferating region in the crypt, we have adapted a method of isolating cells along the crypt-villus axis in fractions.^15,16^ This method allows for separation of gut epithelial cells resulting in fractions enriched for cells from the crypt region (Supplementary Fig. S3) and has been previously validated by IHC and gene array analysis.^21,22^ The early fractions (i.e. 1 and 2) contain cells near the villus tip and while the late fraction (i.e. 3) are enriched in cells from the crypt region. Proteins isolated using this fractionation method were initially evaluated for efficacy. Mice were injected with either 5 mg/kg UCN-01 or DMSO. Twenty-four hours later, the mice were sacrificed, and the jejunum harvested and serially digested. Serial fractions of jejunum epithelium were separated using the Weiser method.^15^ The proliferative marker PCNA was highest in fraction 3 as assessed by western blot analysis of PCNA in extracts from each fraction (i.e. 1–3) and quantitated by densitometry (Fig 3c, d, e) and compared with the fractions from DMSO treated mice (Fig. 3c, d, e). These results are consistent with proliferating crypt cells as confirmed visually by microscopy. Based on these findings, all subsequent analyses were performed on fraction 3 of the mouse gut samples. We analysed key G1 proteins by western blot analysis in fraction 3 samples from UCN-01-treated mice and DMSO controls. Of the positive (cyclin E, cyclin D, CDK4, CDK2) and negative (p21, p27) cell cycle regulators examined in fraction 3 of the gut samples, p21 and p27 levels were modulated, showing increased expression in the UCN-01 treated samples (Fig. 3f and data not shown). p21 and p27 induce cell cycle arrest through binding of CDK1 and/or CDK2. Such binding could result in down regulation of CDK1 or CDK2. Therefore, we also measured the CDK1 and CDK2 activities in these same samples and found that while CDK2 was unchanged (Supplementary Fig. S4), CDK1 was down-regulated following UCN-01 treatment (Fig. 3f). To assure that proteins were not being altered by the fractionation procedure itself, we also assessed CDK2 activity by IHC for phospho-CDK2 in both groups on crypt cells that were not fractionated and found that levels of CDK2 kinase activity was similar in the UCN-01 treated mice as compared with DMSO controls, in agreement with the findings in the serial fractionation process (Supplementary Fig. S4).

### UCN-01 protects normal cells from 5-FU toxicity

**Fig. 4:**
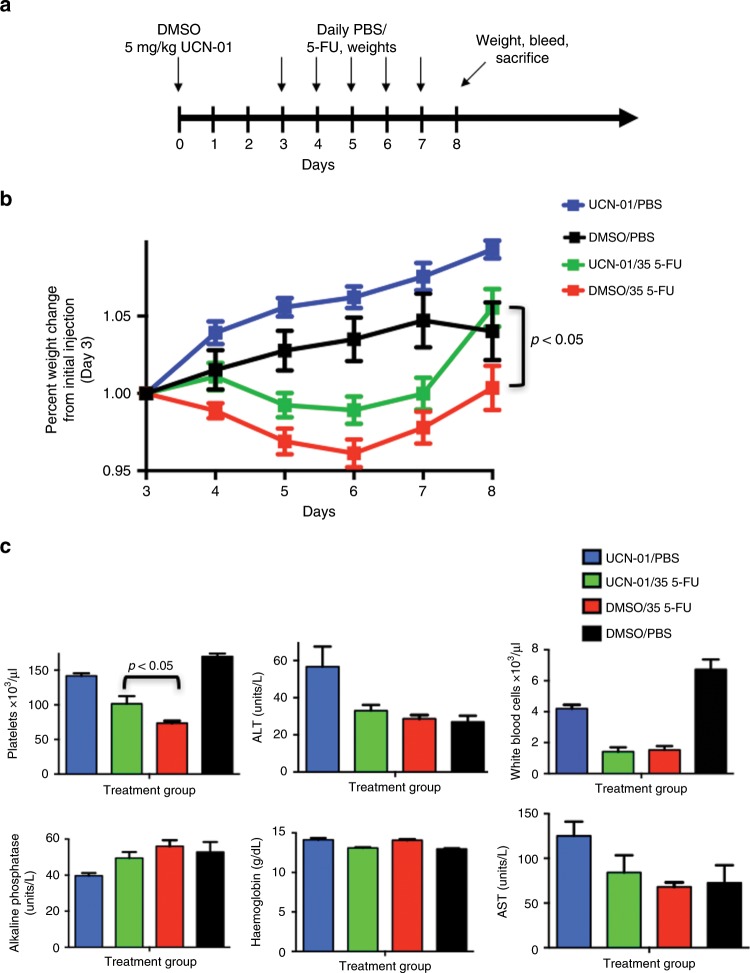
Pre-treatment with UCN-01 improves the toxicity profile for mice treated with 5-FU. **a** Mice were treated with UCN-01 5 mg/kg or DMSO control on day 0 followed by either 5-FU 35 mg/kg or PBS daily on days 3–7 (20 mice total, five mice per group); **b** Weight at day 8 was significantly higher for mice treated with UCN-01/5-FU as compared with DMSO/5-FU (*p* < 0.05); **c** Haematologic parameters and hepatic function was measured across groups and showed significantly higher platelet counts in mice pre-treated with UCN-01 prior to 5-FU at day 8. No difference was observed in hepatic function tests or white blood cell counts between DMSO and UCN-01 pre-treated mice receiving 5-FU.

**Fig. 5:**
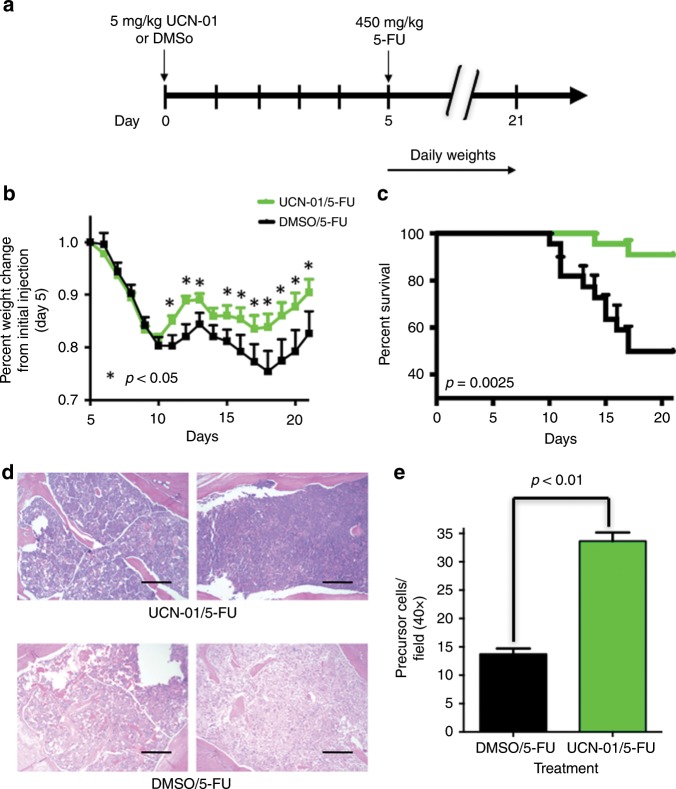
Pre-treatment with UCN-01 protects against chemotoxicity of bolus 5-FU administration. **a** Mice were treated with UCN-01 5 mg/kg or DMSO control on day 0 followed by a single bolus dose of 5-FU 450 mg/kg on day 5 (44 mice total, 22 mice per group); **b** UCN-01 pre-treated mice had significantly less weight loss for days 11–21 (*p* < 0.05); **c** improved overall survival at day 20 (p = 0.0025); and **d**, **e** less myelosuppression, as demonstrated by higher levels of haematologic precursors in sternal tissue at sacrifice on day 21 (*p* < 0.05). The area of haematologic precursor cells was normalised to the total stained area of a 40x section. Five mice per group were evaluated with quantification of five fields per mouse. Scale bar: ×100 magnification.

**Fig. 6:**
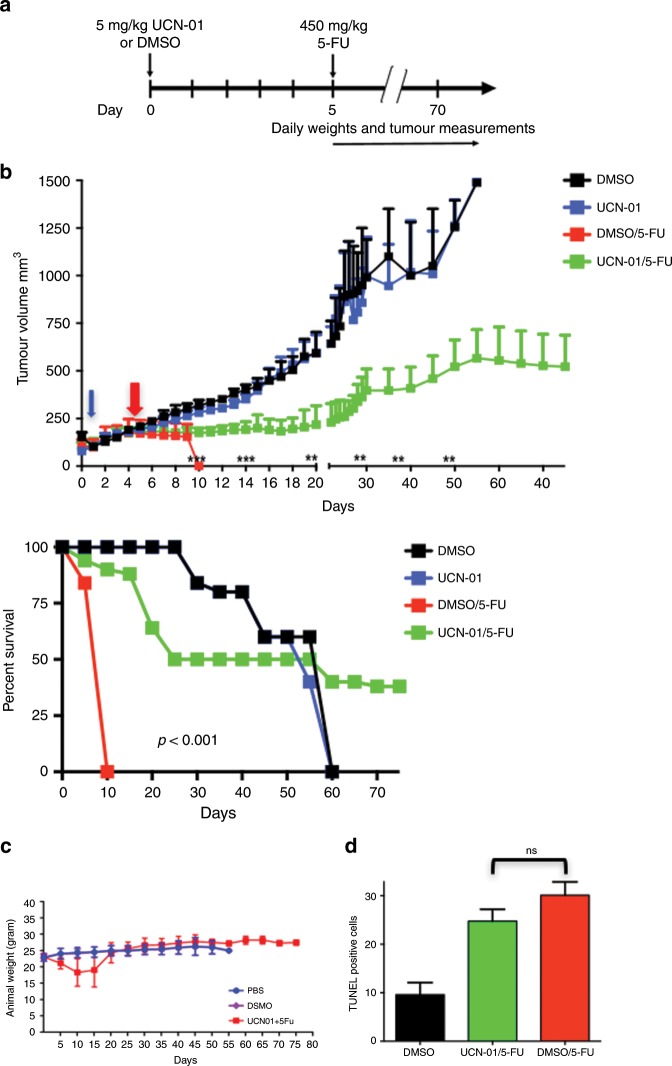
Pre-treatment with UCN-01 in tumour-bearing mice results in decreased tumour volumes and prolonged survival. **a** MDA-MB-468 breast adenocarcinoma tumour-bearing mice were treated with UCN-01 5 mg/kg or DMSO control on day 0, followed by a single bolus dose of 5-FU 450 mg/kg or PBS on day 5. Eight mice were treated per group and experiments repeated three times. **b** All mice in all treatment groups were weighed daily from days 1 to 30, and twice a week between days 30 and 75. Mice pre-treated with UCN-01 (green line) on day 0 (blue arrow) prior to 5-FU on day 5 (red arrow) maintained significant, decreased tumour volumes as compared with DMSO controls (red line), whereas UCN-01 alone (blue line) did not inhibit tumour growth as compared with DMSO (black line). (**p < 0.01, ***p < 0.001 comparing UCN-01/5FU to UCN-01 arms on the indicated dates). **c** Pre-treatment with UCN-01 prior to 5-FU resulted in significantly improved survival (*p* < 0.001). All mice receiving DMSO/5-FU died by day 10 related to treatment toxicity; **c** Weight status was maintained in mice receiving UCN-01 pre-treatment; **d** Apoptosis assessed by TUNEL staining showed no difference between the UCN-01/5-FU and DMSO/5-FU treatment groups, confirming that UCN-01 pre-treatment did not exert significant anti-tumour activity or modulate the cytotoxic effects of 5-FU on the tumour.

Having identified the (i) dose of UCN-01 required to induce a cell cycle arrest and (ii) time post UCN-01 for the resumption of cell proliferation, we next set out to evaluate the potential of UCN-01 as a cytoprotective agent against cytotoxic chemotherapy in non-tumour-bearing (Figs. 4 and 5) and tumour-bearing (Fig. 6) animals. To this end, we initially sought to determine whether the temporary arrest of the normal dividing tissues of the nu/nu nude mouse by UCN-01 pre-treatment improved tolerance and decreased toxicity caused by fluorouracil (5-FU) administration. We further examined if 2 days post UCN-01 treatment is sufficient lead time prior to 5-FU administration to mediate cytoprotection. 5-FU treatment has a well-established toxicity profile, resulting in cellular damage to the gut epithelium, and in patients can yield significant mucositis and myelosuppression.^23,24^ Mice were treated with UCN-01 to induce cell cycle arrest, and 48 h later, were injected with 5-FU (35 mg/kg). 5-FU was administered at varying time points from one to seven days post-UCN-01, and tolerance of this treatment was evaluated by measuring weight, blood markers and survival. Mice were pre-treated with UCN-01 or DMSO control 3 days prior to administration of ip 5-FU or ip PBS daily for 5 days (Schema Fig. 4a). Pre-treatment with UCN-01 resulted in improved weight status in 5-FU treated mice (Fig. 4b). Furthermore, platelet counts were significantly higher in mice receiving UCN-01 at day 8 (*p* = 0.04), however haemoglobin and white blood cell counts were not significantly different between the treatment groups. Hepatotoxicity as assessed by AST, ALT and alkaline phosphatase was unchanged (Fig. 4c). The increase in weight seen in both the UCN-01/5-FU and DMSO/5-FU groups between day 7–8 is attributed to discontinuing 5FU. These results suggested that 2 days of post UCN-01 treatment only yielded partial protection and that 5 days of low-dose 5FU was insufficient to yield a greater degree of weight change. Next, to investigate the protective effects of UCN-01 against bolus administration of 5-FU in non-tumour-bearing animals, 22 mice per group were treated with either UCN-01 or DMSO control 5 days prior to high-dose (450 mg/kg) bolus ip administration of 5-FU (schema Fig. 5a). Mice receiving UCN-01 pre-treatment had improved weight status and survival (91% vs 50% at day 20) as compared with the DMSO controls (Fig. 5b, c). The protective effect of UCN-01 in maintaining normal levels of haematological precursors were also examined in these mice by quantitating the precursor cells in the haematoxylin and eosin stained sternal tissues from surviving mice, harvested at 21 days post DMSO/5-FU or UCN-01/5-FU. Results revealed that UCN-01 pre-treated mice had significantly more precursors than the DMSO mice (Fig. 5d, e). Collectively, the studies in non-tumour-bearing animals suggested that UCN-01 pre-treated mice had significant improvement in tolerance of 5-FU treatment compared with DMSO-pre-treated control mice. However, this improvement is only observed when 5-FU is administered during a window of efficacy, ~3–5 days post UCN-01 treatment. These studies also show that the optimal time for protection from the toxicity of a bolus dose of 5-FU is 5 days post UCN-01.

### Pre-treatment with UCN-01 in tumour-bearing mice results in decreased tumour volumes and prolonged survival

UCN-01 treatment in vivo results in reversible cell cycle arrest and improves tolerance to the cytotoxic effects of 5-FU chemotherapy. To be effective as a cytoprotective strategy, these effects must be specific to normal proliferating cells without affecting tumour cells. Having shown that pre-treatment of non-tumour-bearing mice with UCN-01 can protect the mice against the toxic effect of bolus 5-FU treatment (450 mg/kg) (Fig. 5), we next asked if a similar treatment schedule can be effective in reducing tumour burden, without causing toxicity in mice. We used the human breast adenocarcinoma cell line MDA-MB-468 to evaluate this strategy. Tumour cells were injected into the mammary fat pad of each (*n* = 32) nu/nu nude female mouse and were allowed to establish a measurable tumour (50–100 mm), at which point the mice were randomised into four treatment arms (*n* = 8 mice/arm, schema Fig. 6a): 1) UCN-01 (5 mg/kg) on day 0 followed by PBS control, 2) UCN-01 pre-treatment on day 0 followed by bolus 5-FU (450 mg/kg) on day 5, 3) DMSO pre-treatment on day 0 followed by bolus 5-FU on day 5 and 4) DMSO alone on days 0 and 5 as an untreated control group. These experiments were repeated three times (eight mice per treatment arm per experiment) and mice were followed out to 75 days for tumour assessment and survival analysis. Results revealed that pre-treatment with UCN-01 followed by 5-FU bolus (450 mg/kg) treatment significantly decreased tumour volumes as early as 10 days post UCN-01 treatment as compared with DMSO or UCN-01 alone control groups (Fig. 6b). UCN-01 treatment alone did not inhibit tumour growth as these MDA-MB-468 cell line xenografts have a deregulated G1 to S transition and unresponsive to low dose UCN-01. Pre-treatment with UCN-01 however, significantly prolonged survival of the 5-FU treated mice, such that their survival was comparable to controls (Fig. 6c, compare green to black/blue lines); mice receiving bolus 5-FU without UCN-01 pre-treatment died by day 10 due to treatment-related toxicity (Fig. 6c, red line). Weight status was maintained in mice treated with UCN-01 followed by bolus 5-FU and was not significantly different from PBS and DMSO controls (Fig. 6d). Apoptosis was evaluated by TUNEL staining in tumour tissue and was not significantly different between mice receiving UCN-01 pre-treatment prior to 5-FU as compared with mice receiving 5-FU alone, suggesting that the survival benefits of UCN-01 pre-treatment were not due to any direct anti-tumour effects of UCN-01, but rather due to the improved tolerance of the bolus 5-FU administration (Fig. 6c, d and Fig. 5).

## Discussion

In this study, we tested the hypothesis that UCN-01 is a cytoprotective agent temporarily inhibits the cell cycle progression of normal proliferating cells and improving the survival and health of mice receiving high-dose chemotherapy. Our in vivo findings suggest that UCN-01 meets both criteria for efficacy as a cytoprotective strategy: (1) the arrest of normal small bowel epithelial cells was reversible, and (2) the cell cycle arrest was specific to normal proliferating cells and did not protect tumour cells from the cytotoxic effects of 5-FU chemotherapy. Using a tumour-bearing mouse model, we have also shown that pre-treatment with UCN-01 prior to chemotherapy improves tolerance and prolongs survival.

Alternate strategies for cytoprotection of normal cells against cytotoxic chemotherapy include short-term starvation/fasting, the use of CDK4/6 inhibitors, or exploiting p53 as a means to differentially arrest normal vs tumour cells. Fasting has been shown to induce differential stress resistance which protects normal cells but not cancer cells against chemotherapy in both in vitro studies and mouse models. For example, short-term starvation of mice prior to administration of etoposide resulted in reduced chemotoxicity and improved survival in NXS2 murine neuroblastoma mouse model.^25^ More recently, Piwnica-Worms and colleagues have shown that short-term fasting can protect mice from DNA damaging agents by protecting small intestinal stem cell populations and preserving intestinal architecture.^26,27^ Fasting resulted in significantly reduced BrdU incorporation in small intestinal stem cells, indicating that stem cells responded to fasting by reducing proliferation and likely inducing G1 arrest. Specific CDK4/6 inhibitors have also been used to induce cell cycle arrest as a cytoprotective strategy of normal cells against both chemotherapy and radiation. Palbociclib (PD-0332991) has been shown to reduce carboplatin-induced myelosuppression by protecting hematopoietic progenitor cells in WT mice and was effective in decreasing thrombocytopenia an Rb-deficient mouse model of breast cancer.^28^ Another recent study examined the use of palbociclib in reducing radiation-induced intestinal injury in mice.^29^ Pre-treatment with the CDK4/6 inhibitor blocked crypt cell proliferation via G1 arrest resulting in decreased apoptosis, enhanced stem cell survival, and improved crypt regeneration. Lastly, exploiting differences in cells with mutant vs. wild-type p53 poses another strategy for the protection of normal cells against microtubule inhibitors. Activation of p53 with agents such as low-dose actinomycin D,^30^ MDM2 antagonists,^31^ or other low-dose DNA damaging agents^32^ have been shown to induce selective p53 dependent G2 arrest in normal cells without arresting tumour cells, thus permitting selective killing of p53 deficient tumour cells. UCN-01 has similarly been used to release cancer cells with mutant p53 from mitosis, thus increasing toxicity specifically to cancer cells with mutant p53.^33^ This may explain in part not only why UCN-01 can protect normal cells but also has the ability to potentiate treatment of cancer cells.

Enthusiasm for targeted therapies and immunotherapies in the treatment of cancer is at an all-time high, however cytotoxic chemotherapy remains the standard of care and in many cases, is the most effective treatment for solid tumours. Supportive agents that reduce chemotoxicity by promoting protection or regeneration of normal cells are routinely used to maintain dose intensity and decrease therapy-related adverse events. Examples include the use of granulocyte colony stimulating factor (G-CSF) to allow for dose-dense administration (every 2 weeks rather than 3 weeks) of adjuvant chemotherapy in breast cancer patients, which is associated with improved overall and disease-free survival.^34,35^ These agents are also used for primary and secondary prevention of febrile neutropenia in at-risk patients.^36^ Other examples include the use of either oral cryotherapy^37^ or the recombinant human keratinocyte growth factor palifermin^38,39^ to prevent oral mucositis in patients receiving bolus administration or high-dose chemotherapy. Selective, reversible G1 arrest of normal cells poses a novel strategy to enhance tolerance and potentially allow for dose escalation or increased dose intensity of cytotoxic chemotherapy. While fasting has shown efficacy in preclinical models, concerns have been raised regarding the long-term feasibility of dietary restriction or fasting in cancer patients undergoing treatment. For the CDK4/6 inhibitor palbociclib, cell cycle arrest is not specific to normal cells and reversibility has not been shown. At the doses used clinically, the addition of palbociclib to hormonal therapy in breast cancer patients resulted in significantly increased neutropenia.^40,41^ Drugs such as UCN-01, which can differentially induce G1 arrest in normal versus tumour cells at differing doses have the greatest therapeutic potential. While prior clinical studies of high-dose UCN-01 focusing on anti-tumour activity were largely ineffective,^18,42,43^ when given at a low dose at a specified interval prior to administration of chemotherapy, these agents may allow for dose escalation of cytotoxics while minimising chemotoxicity. Alternative agents that can induce reversible G1 arrest such as Chk1 inhibitors warrant further investigation as a cytoprotective strategy to increase the therapeutic index of cytotoxic chemotherapy regimens.

In summary, we show that treatment of mice with low-dose UCN-01 induces a reversible, post-mitotic G1 arrest in normal cells of the small bowel and that this arrest improves tolerance to bolus 5-FU in non-tumour-bearing mice. In addition, in tumour-bearing mice we show that pre-treatment with low-dose UCN-01 prior to 5-FU decreased chemotoxicity and allowed for dose escalation of 5-FU to enhance its therapeutic efficacy.

## Supplementary information

Supplemental data and Figure legends-with scale bars

## Data Availability

All data generated or analysed during this study are included in this published article [and its supplementary information files].
