# Supplementary material for: Specific, reversible G1 arrest by UCN-01 in vivo provides cytostatic protection of normal cells against cytotoxic chemotherapy in breast cancer
Source: Br J Cancer. 2020 Jan 16;122(6):812–22. doi: 10.1038/s41416-019-0707-z (PMC7078276; doi:10.1038/s41416-019-0707-z)
Supplement: Supplementary file 1 — Supplemental data and Figure legends-with scale bars [file 41416_2019_707_MOESM1_ESM.pdf]

Specific, reversible G1 arrest by UCN-01 *in vivo* provides cytostatic protection of normal cells against cytotoxic chemotherapy

Benjamin B. Mull, J. Andrew Livingston, Nalini Patel, Tuyen Bui, Kelly Hunt, and Khandan Keyomarsi

SUPPLEMENTARY INFORMATION

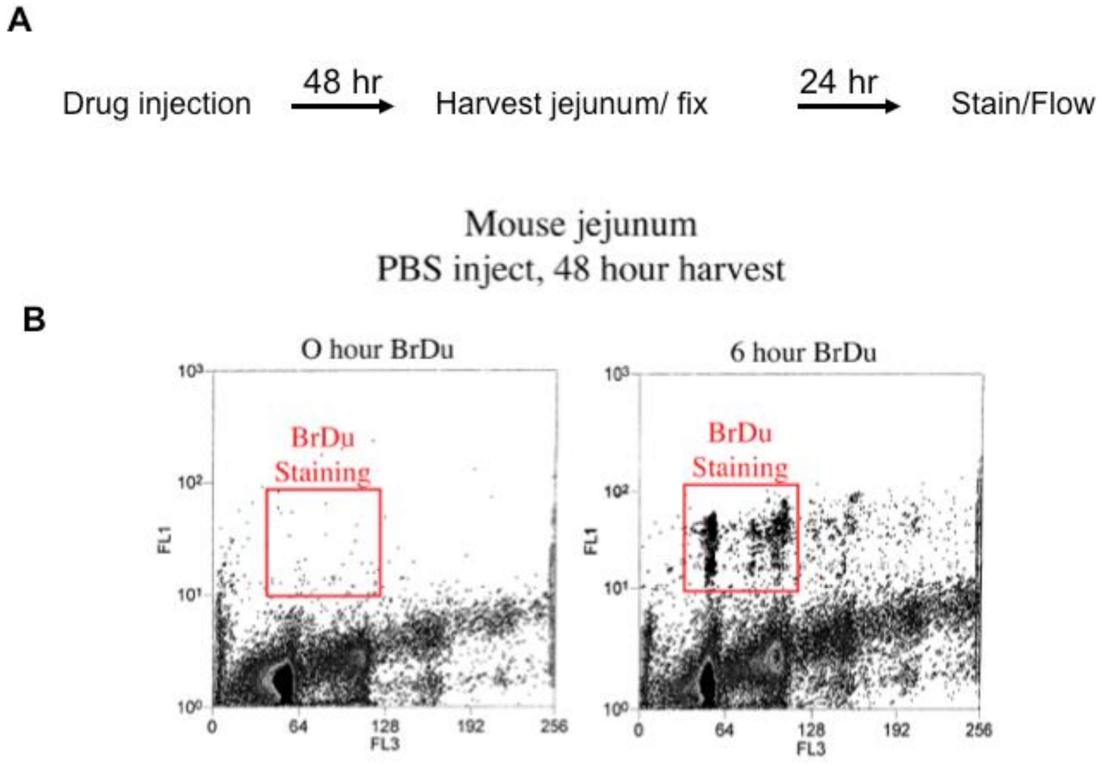

Supplementary Figure S1. Assessment of BrdU labeling in mouse jejunum. (A) In initial studies, mice were treated with either drug or control and sacrificed at 48 hours post-injection, jejunal tissues was harvested and fixed, and subsequently analyzed by either IHC or flow cytometry 24 hours later; (B) Mice receiving PBS control were injected with BrdU at varying time points prior to sacrifice to evaluate BrdU labeling. BrdU labeling of normal cells was greatest when BrdU was given 6 hours prior to sacrifice.

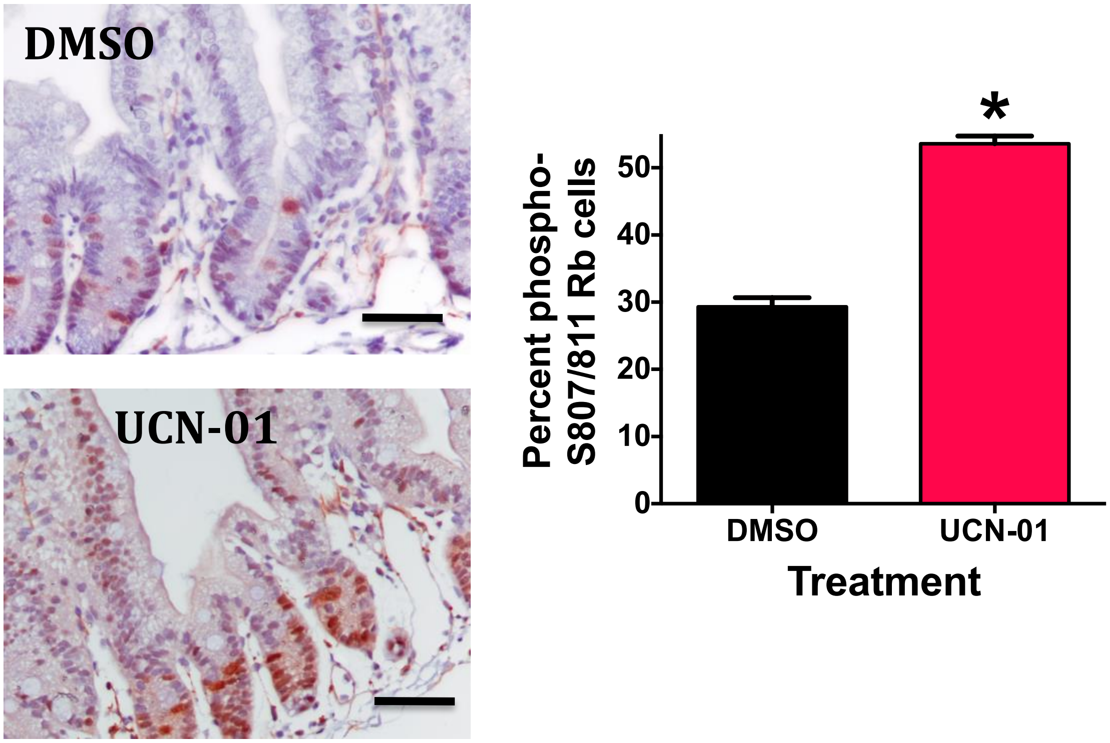

Supplementary Figure S2. Phosphorylated-Rb increases in crypt cells following UCN-01 treatment. Mice were treated with DMSO control (top panel) or UCN-01 5mg/kg (bottom panel) and sacrificed at 24 hours. Phosphor-Ser807/811 Rb staining of jejunal crypt cells was significantly higher in UCN-01 treated mice (5 mice per group, 10 crypts per mouse analyzed). Scale bar: X 400 magnification.

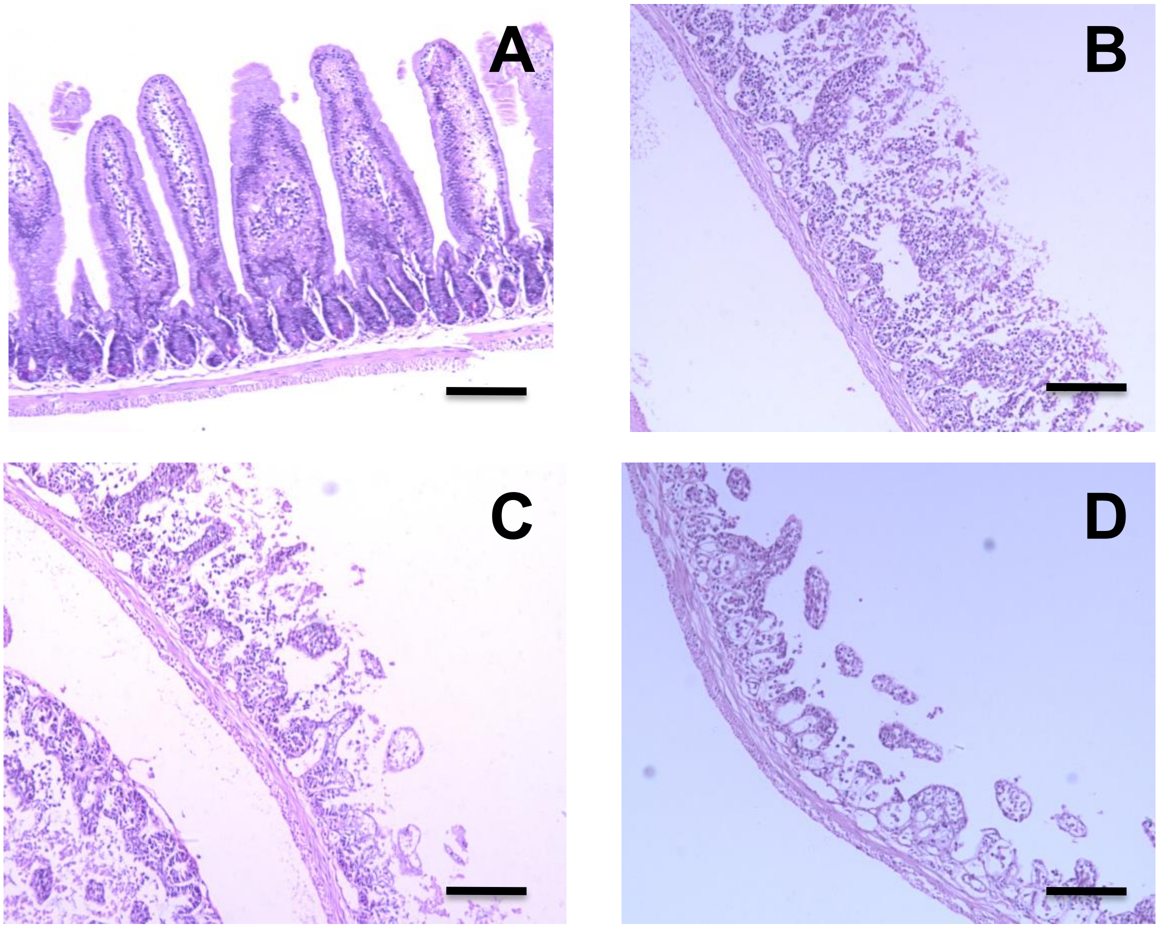

Supplementary Figure S3. Serial fractionation of small bowel epithelium. H&E sections of everted small bowel at the 4 stages of the serial fractionation procedure demonstrates continual digestion of epithelial cells from the villus tip downward into the crypt. Samples were processed to display tissue at time zero (A); following 24 minutes incubation, fraction 1 cells removed (B); 60 minutes, fraction 2 cells removed (C); and 120 minutes, fraction 3 cells removed (D). This procedure allows for the enrichment of crypt cells in fraction 3. Scale bar: X 150 magnification.

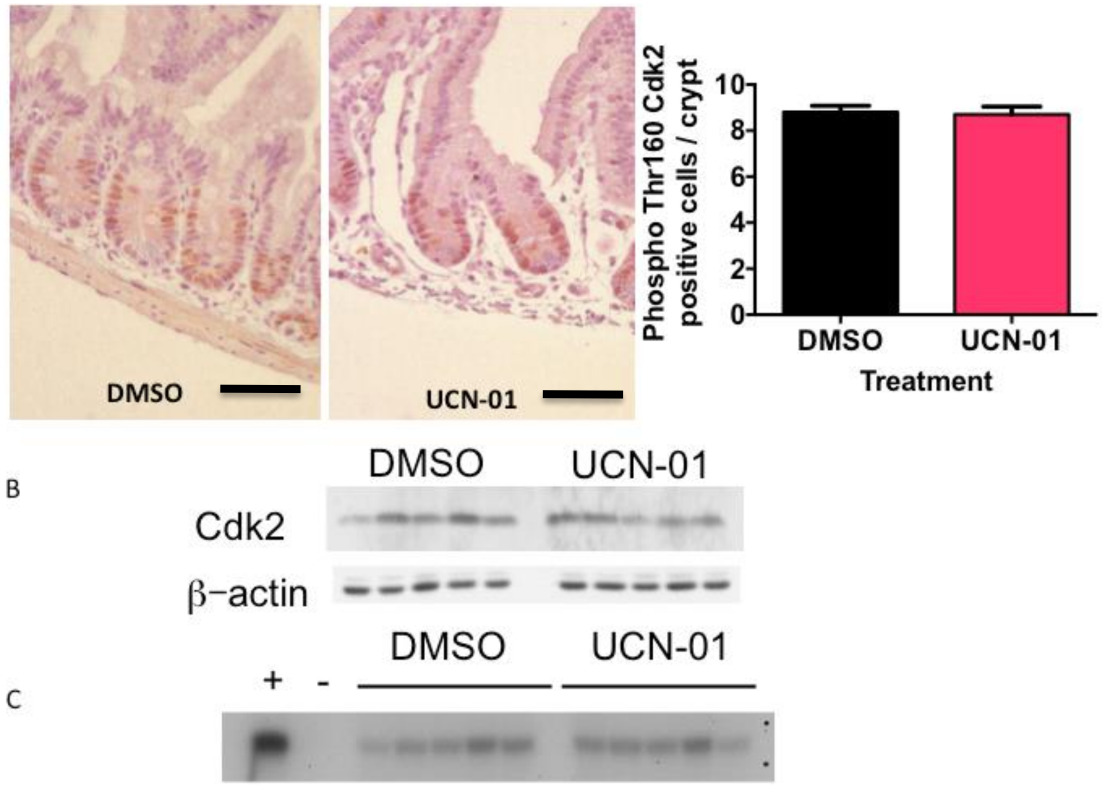

Supplementary Figure S4. CDK2 activity is unchanged following UCN-01 treatment. (A) Representative IHC for active (phosphor-Thr160) CDK2 in mouse jejunum 24 hours after DMSO or 5mg/kg UCN-01. UCN-01 treatment resulted in significant change in phosphor-CDK2 in jejunal crypt cells (5 mice per group, 10 crypts per mouse analyzed); Western blot of mouse jejunum crypt fractions showed no significant difference (densitometry not shown) in either CDK2 (B) or CDK2 kinase (C) between UCN-01 treated mice and DMSO controls. MCF-7 cell lysate is used as positive control for CDK2 kinase. Scale bar: X 400 magnification.
